# Seroprevalence and risk factors for *Neospora* spp. infection in equine in Egypt

**DOI:** 10.1038/s41598-023-47601-y

**Published:** 2023-11-19

**Authors:** Ayed Alshammari, Hattan S. Gattan, Mohamed Marzok, Abdelfattah Selim

**Affiliations:** 1 Department of Biology, College of Science, University of Hafr Al-Batin, Hafr Al-Batin, Saudi Arabia; 2 Department of Medical Laboratory Sciences, Faculty of Applied Medical Sciences, King AbdulAziz University, Jeddah, Saudi Arabia; 3 Special Infectious Agents Unit, King Fahad Medical Research Center, King AbdulAziz University, Jeddah, Saudi Arabia; 4 Department of Clinical Sciences, College of Veterinary Medicine, King Faisal University, 31982 Al-Ahsa, Saudi Arabia; 5 Department of Surgery, Faculty of Veterinary Medicine, Kafr El Sheikh University, Kafr El Sheikh, Egypt; 6 Department of Animal Medicine (Infectious Diseases), Faculty of Veterinary Medicine, Benha University, Toukh, 13736 Egypt

**Keywords:** Microbiology, Diseases, Risk factors

## Abstract

*Neospora* infections in equine are associated with reproductive disorders and neurological diseases. Nevertheless, Egypt has no epidemiological information on this parasite in equine. This study determined the prevalence of *Neospora* spp. infection in 325 equines from three Egyptian governorates located at Northern Egypt using cELISA. The prevalence of antibodies against *Neospora* spp. was 19% (95% CI: 14.09–25.05) in horse, 34.1% (95%CI: 24.92–44.69) in donkey and 26.7% (95% CI: 15.97–41.04) in mule. In comparison to horse, donkey had a considerably higher chance of contracting *Neospora* spp. infection (OR = 1.80, 95% CI: 0.78–4.13; *P* = 0.016). The risk was also higher in freely grazing animals (OR = 3.49, 95% CI: 0.95–12.78; *P* = 0.059). Moreover, yearling animals (12–24 months) (OR = 5.03, 95% CI: 1.51–16.80; *P* = 0.009) and those with natural breeding (OR = 11.80, 95% CI: 3.24–42.99, *P* < 0.0001) and a history of early abortion (OR = 7.04, 95% CI: 3.01–16.47; *P* < 0.0001) also showed a greater risk of seropositivity. The risk of *Neospora* infection increased significantly in equines contacted with dogs (OR = 5.16, 95% CI: 1.76–15.10; *P* = 0.003). This the first serological study to determine the seroprevalence of *Neospora* spp. in equine in Egypt. Further studies are necessary to identify the species of *Neospora* and to understand the role of above-mentioned risk factors in spreading of infection.

## Introduction

Neosporosis is one of the most important diseases that affects cattle and dogs and has a global distribution. The disease caused by *Neospora caninum*, which is one of the major cause of abortion in cattle and cause significant financial losses in dairy industry^1^. *N. caninum* is intracellular obligate protozoan belongs to the phylum Apicomplexa. Many host species have been reported to be infected with *N. caninum*, with dogs and related canids are regarded to be a definite host, whereas *N. hughesi* has only found in equines and its definitive host has not yet been identified^2,3^.

The transmission of *Neospora* spp. can be occurred either vertically or horizontally. According to a Brazilian study, transplacental transmission of *Neospora* occurs in 34.8% of borned foals from seropositive mares which showed anti-*Neospora* pre-colostral antibodies^4^. Despite transplacental transmission of *Neospora* spp. in equines^5,6^, the role of *Neospora* and transplacental transmission route in equine abortion are still not fully understood^7^. In equines, the reproductive disorders and neonatal mortalities associated with *N. caninum* infection, while the *N. hughesi* causes neurological affections^8,9^.

Nonetheless, *Neospora* infection in intermediate hosts typically goes undetected because of the absence of symptoms and the exposure is determined by the existence of anti-*Neospora* antibodies. The most popular technique for determining the distribution and epidemiology of neosporosis in susceptible animals is detection of antibodies against *Neospora* spp. in serum samples^10^. However, as serological discrimination between these two species is likely not attainable, the possibility of cross-reaction between *N. caninum* and *N. hughesi* in serological testing complicates the interpretation of serological surveys results^11^.

Numerous countries have been reported *Neospora* spp. serologically in equines. The *Neospora* antibodies were found in 2% of equine serum samples from South Korea^12^, 11.5% of samples from North America^13^, 2.5% of samples from South America^14^, 23.8% of samples from New Zealand^2^ and 23% of samples from France^15^.

In a serological study on horses in Israel, the seroprevalence of *Neospora* spp. was 11.9% in apparent healthy horses but it was significantly higher among horses had neurological symptoms (21.2%) or in mares suffered from abortion (37.5%)^5^. In addition, other studies were conducted in Brazil to investigate antibodies against *Neospora* spp., Whereas Villalobos et al.^6^ found a prevalence of 15.1% in animals raising in So Paulo state but was 2.5% in serum samples from ten federal states as reported by Hoane et al.^14^.

Animals infected with *N. caninum* have been identified using a variety of laboratory procedures. In order to identify specific antibodies in the sera of infected animals, a range of serology procedures are employed, including the indirect fluorescent antibody test (IFAT), direct agglutination tests, immunoblotting (IB), and ELISAs. Moreover, *N. hughesi* can be differentiated from *N. caninum* by DNA biotechnology^16–19^. We used ELISA to establish the long-term stability of high antibody levels against the parasite in equine^20^. Serologic testing has a lower cost advantage than other tests. ELISA is the most suited serologic technique for high throughput screening of antibodies to this parasite^21^.

In Egypt, the prevalence and associated risk factors for *Neospora* infection have been studied in domestic ruminants. The prevalence rates were ranged between 8.6 and 36.1% in sheep^22,23^, 10.9% in camels^24^ and 20.43–24.6% in dairy cattle^25,26^. However, there is a lack of epidemiological information about the disease in equine.

Therefore, the goal of the study was to determine the seroprevalence of *Neospora* spp. in equine and to identify the associated risk factors for *Neospora* infection in equine in three Egyptian governorates.

## Materials and methods

### Study area

The study was conducted on equine raising in three governorates namely Giza, Kafr ElSheikh and Qalyubia which situated at North of Egypt. The selected governorates are distinguished by having a high equine population where equine is used in tourism around the pyramid area in the Giza governorate. In addition, equine is mostly utilized in breeding and droughting in other governorates, including Kafr ElSheikh and Qalyubia.

Giza governorate situated at west of Nile and southwest to Cairo. Giza's climate is hot and dry (Köppen: BWh); the average annual temperature is 20 °C, but the summer months can reach a maximum of 40 °C. There is also rare precipitation throughout the year. On the other side, the governorates of Kafr ElSheikh and Qalyubia located at Egypt’s Nile Delta, which are distinguished by a year-round average temperature of 28 °C and by cold temperatures and minimal precipitation in the winter.

### Study design

Across sectional study was performed during January to December 2022. Blood samples from 325 asymptomatic equine were taken by convenience from three governorates in Egypt: 195 horses, 85 donkey, and 45 mules. These animals were asymptomatic and selected randomly from the three governorates under the study.

Blood sample (5 mL) were taken from jugular vein of each animal using vacutainer, which transferred in icebox to veterinary Diagnostic Laboratory, Faculty of Veterinary Medicine, Benha University. Sera were separated by centrifugation at 3000 × g for 10 min, and then stored at − 20 °C until serological examination. During sampling, data of each examined animal were taken including equine type (horse, mule and donkey), age (12 months, 12–24 months, and > 24 months old), sex (male and female) and location, Table 1. A questionnaire was prepared to collected data of other management factors like type of animal management or breeding, history of abortion and contact with dogs.

**Table 1:** Descriptive analysis for factors used to determine the seroprevalence of *Neospora* infection in equine.

Variable	Category	Number of tested animal	Distribution (%)
Locality	Giza	110	33.8
	Kafr ElSheikh	120	37
	Qalyubia	95	29.2
Species	Horse	195	60
	Donkeys	85	26.2
	Mules	45	13.8
Sex	Male	110	33.8
	Female	215	66.2
Age	< 12 months	52	16
	12–24 months	186	57.2
	> 24 months	87	26.8
Management	Grazing	141	43.4
	Stable	50	15.4
	Mixed	134	41.2
Type of breeding	Natural breeding	150	69.7
	artificial insemination (AI)	65	30.2
History of abortion	No history of abortion	140	65.11
	Early abortion	34	15.8
	Late abortion	41	19.1
Contact with dogs	Yes	241	74.2
	No	84	25.8

### Serological analysis

All sera were examined for the existence of antibodies against *Neospora* spp. using a commercial competitive inhibition enzyme-linked immunosorbent assay, (cELISA, VMRD, Inc., Pullman, WA, USA). Serological testing and evaluation were carried out in accordance with the manufacturer's recommendations. The optical densities (ODs) were measured using ELISA plate reader AMR-100 (AllSheng, Chnia) at 650 nm. This test has a sensitivity and specificity of 91.4% and 99.4%, respectively^27^. Moreover, the manufacturer provided negative and positive control with cELISA kit. After calculating the percent inhibition, samples had an inhibition of less than 30% considered positive.

### Statistical analysis

Data were analyzed using the statistical SPSS software version 24 (IBM, SPSS Inc., Chicago, IL, USA). Data on seroprevalence of *Neospora* spp. were statistically evaluated, taking into consideration factors like location, gender, age, species, type of breeding, type of management, history of abortion, and contact with dogs. Using the Chi-square test, the association between neosporosis and other risk variables was evaluated. The results considered significant in case of *P* value less than 0.05. The multivariate logistic regression model was used to assess the independent risk factors of each explanatory variable for all variables that had a *P* value in the univariate analysis of less than 0.25^28^. The occurrence odds ratio (OR) and associated 95% confidence interval (CI) were calculated by multivariate logistic regression^29–32^. The goodness-of-fit of the model was evaluated using the Hosmer–Lemeshow statistic^33^.

### Ethics approval and consent to participate

The study protocol followed the guidelines of the ethical committee of the Faculty of Veterinary Medicine at Benha University, which complies with all Egyptian regulations regarding research and publication. Serum samples were collected and handled in accordance with the Committee's Animal Ethical Standards and Guidelines. The procedures of present study were conducted in accordance with the ARRIVE recommendations.

## Results

Among the 325 equine samples, 78 (or 24%) tested positive for IgG antibodies to *Neospora* spp. The prevalence rate was statistically differed (*P* < 0.05) between various equine hosts, it was 19% in horse, 34.1% in donkey, and 26.7% for mule. However, no-statistical difference (*P* > 0.05) was observed between sex. Moreover, there were statistically different in seropositivity for *Neospora* spp. between age groups (*P* < 0.05). Antibodies against *Neospora* spp. were found in 7.7% of foals, 31.2% of yearlings, and 18.4% of adults, Table 2.

**Table 2:** Univariable analysis for factors used to determine the seroprevalence of *Neospora* infection on equine.

Variable	Total tested animal	No of positive	No of negative	% of positive	95% CI	Statistic
Locality						
Giza	110	25	85	22.7	15.9–31.4	χ^2^ = 2.166 df = 2 *P* = 0.339
Kafr ElSheikh	120	34	86	28.3	21.04–36.97	
Qalyubia	95	19	76	20.0	13.19–29.14	
Species						
Horse	195	37	158	19.0	14.09–25.05	χ^2^ = 7.646 df = 2 *P* = 0.022*
Donkeys	85	29	56	34.1	24.92–44.69	
Mules	45	12	33	26.7	15.97–41.04	
Sex						
Male	110	24	86	21.8	15.12–30.42	χ^2^ = 0.434 df = 1 *P* = 0.510
Female	215	54	161	25.1	19.79–31.32	
Age						
< 12 months	52	4	48	7.7	3.03–18.17	χ^2^ = 14.343 df = 2 *P* = 0.001*
12–24 months	186	58	128	31.2	24.96–38.16	
> 24 months	87	16	71	18.4	11.65–27.8	
Management						
Grazing	141	48	93	34.0	26.73–42.19	χ^2^ = 16.366 df = 2 *P* < 0.0001*
Stable	50	4	46	8.0	3.15–18.84	
Mixed	134	26	108	19.4	13.6–26.91	
Type of breeding						
Natural breeding	150	51	99	34	26.9–41.9	χ^2^ = 20.819 df = 1 *P* < 0.0001*
Artificial insemination (AI)	65	3	62	4.6	1.58–12.72	
History of abortion						
No history of abortion	140	18	122	12.9	8.29–19.41	χ^2^ = 44.833 df = 2 *P* < 0.0001*
Early abortion	34	23	11	67.6	50.58–80.87	
Late abortion	41	13	28	31.7	19.57–46.99	
Contact with dogs						
Yes	241	71	170	29.5	24.06–35.5	χ^2^ = 15.243 df = 1 *P* < 0.0001*
No	84	7	77	8.3	4.09–16.21	
Total	325	78	247	24.0	19.68–28.93	

*The result is significant at *P* < 0.05.

The management of equine showed significant impact (*P* < 0.05) on seroprevalence of *Neospora* spp., where only 8% of equine maintained in stables tested positive for antibodies to *Neospora* spp., compared to 34% of equine that were allowed to graze in pasture.

We evaluated the effect of breeding to understand its role in prevalence of antibodies to *Neospora* spp. We found that the seroprevalence was 4.6% in equine that had artificial insemination and 34% in those had natural breeding. The seroprevalence was significantly influenced by the type of breeding, Table 2.

For animals with and without a history of abortion, there was a substantial difference in seropositivity (*P* < 0.0001) between the two groups. The history of abortion in 36 female animals was recorded. The seropositivity rate was found higher in equine (67.6%) with early abortion history than animals with no abortion history (12.9%), Table 2.

The multivariable logistic regression analysis was tabulated in Table 3. The results revealed that the risk of *Neospora* spp. infection in mule and donkey was two times higher than in horse. Yearling equine of 12–24 months age was five times (OR = 5.03, 95% CI: 1.51–16.80) more likely to be infected than foals. Moreover, grazing horses with history of early abortion were three and seven times more likely to be infected when compared with equine kept in stable or equine with no history of abortion. Additionally, natural breeding and contact with dogs in examined equine increased risk of *Neospora* spp. infection eleven and five times more compared to AI and absence of dogs.

**Table 3:** Variables associated with *Neospora* infection in multivariable logistic regression analysis.

Variables	B	S.E	OR	95% CI for OR		P value
				Lower	Upper	
Species						
Donkeys	0.585	0.424	1.80	0.78	4.13	0.016
Mules	0.692	0.494	2.00	0.76	5.26	0.016
Age						
12–24 months	1.616	0.615	5.03	1.51	16.80	0.009
> 24 months	0.890	0.664	2.43	0.66	8.95	0.018
Management						
Grazing	1.249	0.663	3.49	0.95	12.78	0.059
Mixed	1.192	0.679	3.30	0.87	12.46	0.079
Type of breeding						
Natural breeding	2.468	0.659	11.80	3.24	42.99	< 0.0001
History of abortion						
Early abortion	1.952	0.433	7.04	3.01	16.47	< 0.0001
Late abortion	1.172	0.471	3.23	1.28	8.13	0.013
Contact with dogs						
Yes	1.641	0.548	5.16	1.76	15.10	0.003

B, logistic regression coefficient; SE, standard error; OR, odds ratio; CI, confidence interval.

## Discussion

Neosporosis is one of the parasite diseases that affects domestic animals and poses a risk to the public's health in addition to inflicting severe economic losses^34,35^. *Neospora* spp. infections either subclinical or clinical forms have been reported horses from numerous countries across the world^36–38^. In Egypt, many animal species, including cattle, sheep, goats, and camels, have been found to be infected with *Neospora*^23,24^. However, to our knowledge, no earlier research has identified infections with *Neospora* spp. in equine in Egypt. Therefore, the present study aims to investigate the presence of antibodies against *Neospora* spp. in equine in three Egyptian governorates to improve the epidemiology of the disease in these areas and to determine the risk factors that infection poses.

The overall findings of the current study show that 24% of equines in Egypt had antibodies against *Neospora* spp., which are consistent with the stated rate (24%) in horses from the Czech Republic^39^.

In comparison to the prevalence rates of diseases among horse in earlier studies, the seropositive rate of *Neospora* infection in horse (19%) detected in the current study is relatively high by comparison with other studies from Brazil. The prevalence rate was 2.5% in examined horse from ten different Brazilian states according to Hoane et al.^14^, while it was 10.3% among horse from the state of So Paulo^6^ and 4.1% in horse from two areas of Santa Catarina state^40^.

On the other hand, reports from other countries indicate higher prevalence rates. A prevalence of 34% was reported among Chilean horses by Patitucci et al.^41^. For horse of various ages in Italy, Ciaramella et al.^42^ reported a prevalence of 28%. In contrast, animals from Costa Rica were found to have a prevalence rate of 3.5%^43^.

Additionally, when indirect fluorescent antibody test (IFAT) was used, it was discovered that horses from various Italian regions had a prevalence of 28%^42^ and 10%^44^, whereas horses from Israel had a prevalence of 12%^5^. In France, a direct agglutination test on horses yielded a frequency of 23%^45^.

Furthermore, the antibodies against *Neospora* spp. were detected in 34.1% of donkey and 26.7% of mule using cELISA, which is consistent with the percentages seen in donkeys and mules from southern Punjab, Pakistan^27^, it was 32.5% and 26.9%, respectively. This high seropositivity when compared to the 2% and 0.4% prevalence rate of *Neospora* spp. found in donkeys from the northeastern part of Brazil^46,47^.

Another study in Mexico detected antibodies against *N. hughesi* antigen 0.8% of the investigated donkeys^48^. In addition, the seroprevalences for *Neospora* spp. found high among donkeys from Iran (52%) using the agglutination test, Pakistan (32.6%) based on ELISA and in China (9.5%) and Italy (11.8%) using cELISA^27,49–51^.

According to previous literatures, it is clear that the prevalence of *Neospora* spp. in equine varied significantly between and even within countries. These variances may result from the use of different serological tests in each study, the testing methodologies' limitations, non-standard controls, and the applied cut-off points^2,17,23,52–56^. Moreover, *Neospora* spp. seroprevalences may be influenced by the study design, sample collection criteria, varying amounts of exposure to various risks, and protective factors for infection or sickness, which makes it challenging to compare the findings from diverse studies^36,57,58^.

*Neospora* spp. seroprevalence varied significantly between age groups and the highest seroprevalence was observed among equine of 12–24 months age, according to the current study. This finding comes in agreement with the earlier study from Israel^5^, which reported a significant greater seroprevalence to *Neospora* spp. in older horses due to an increased arisk of exposure via horizontal spreading over time. In addition, *Neospora* spp. seroprevalence increased non-significantly (22.2% and 27.2%) between the age groups of 1–10 and 11–20 years old, respectively, in a Turkish study^59^. Contrary to our findings, Bártová et al.^60^ observed that there was no significant difference between equine age groups and the prevalence rose with equine age, ranging from 15.4% in foals to 26.6% in yearlings, indicating postnatal transmission.

In the line of previous results of Villalobos et al.^6^ and Talafha et al.^61^, there was no significant difference (*P* > 0.05) between both sexs. The current findings revealed higher prevalence in females which attributed to females may be more susceptible to *Neospora* spp. infection due to a worse immune response during different times of their life as aresult of stress of pregnancy and lacation, which causes an immunosuppression and increases their vulnerability to infection^62,63^.

Concerning to feeding type of examined equids, the present results were consistent with previous findings of Nazir et al.^27^, where the *Neospora* spp. seroprevalence increased significantly among grazing animals. This could be explained by horizontal transmission of *Neospora* spp. among equids because it is considered as main rout of transmission and increase risk of infection in equids^5^.

The current findings revealed that the natural breeding increased the risk of infection in equine when compared to AI, which was consistent was findings of de Araújo Valença et al.^64^. This explain the vertical transimission play an vital role in transmission of *Neospora* spp. in equine^4^.

In comparison to female equines without a history of abortion, the prevalence of *Neospora* spp. was significantly (*P* < 0.05) greater in females with history of abortion. However the evidence is rather scant compared to cattle, where *N. caninum* has been consistently shown to be a major cause of abortion, *Neospora* species may play a role in equine abortions^6^. Our results are consistent with other researches in which a population of mares with reproductive problems had high percentage of anti-*Neospora* spp. antibodies^45^.

The results demonstrated that the equines contacted with dogs had high signifcant prevalence rate for *Neospora* spp. when comapred with other animals. These results are consistent with those that reported by de Araújo Valença et al.^64^. The existence of seropositive dogs around the investigated equids may indicate that the animals were exposed to the parasite, increasing the risk of oocyst contamination of food or water and subsequent horizontal transmission^8,43^.

The current study's limitations include the bias in sample collection for possible representation of the examined governorates or equine population and the diagnostic approach since it can identify the *Neospora* spp. However, we were unable to determine the species of *Neospora*. As a result, more research is required for large-scale screening and determining *Neospora* species in Egyptian equines.

## Conclusion

This is the first instance of *Neospora* spp. being found in Egyptian equines. Several risk factors are associated with *Neospora* spp. infections like age, species, management, type of breeding, history of abortion and contact with dogs. The findings demonstrate that *Neospora* spp. is a contributing factor in abortions in Egyptian mares. Thus, the prevention and control program should be implemented to prevent the spreading of the diseases among equine and economic losses as a result of abortion. Additional epidemiological researches for molecular detection of parasite DNA in tissue from placentas or aborted fetus to identify *Neospora* spp. are necessary.

## Data Availability

All data generated or analysed during this study are included in this published article.
